# Comparison of Degradation Behavior of Newly Developed Encapsulation Materials for Photovoltaic Applications under Different Artificial Ageing Tests

**DOI:** 10.3390/polym13020271

**Published:** 2021-01-15

**Authors:** Chiara Barretta, Gernot Oreski, Sonja Feldbacher, Katharina Resch-Fauster, Roberto Pantani

**Affiliations:** 1 Polymer Competence Center Leoben GmbH, Roseggerstrasse 12, 8700 Leoben, Austria; gernot.oreski@pccl.at (G.O.); sonja.feldbacher@pccl.at (S.F.); 2 Institute of Material Science and Testing of Plastics, University of Leoben, Otto Glöckl Straße 8, 8700 Leoben, Austria; katharina.resch-fauster@unileoben.ac.at; 3 Department of Industrial Engineering, University of Salerno, Via Giovanni Paolo II, 84084 Fisciano, Italy; rpantani@unisa.it

**Keywords:** ageing, degradation, encapsulant, ethylene vinyl acetate, oxidation, photovoltaic, polyolefin encapsulant

## Abstract

The main focus of this work is to investigate the degradation behavior of two newly developed encapsulants for photovoltaic applications (thermoplastic polyolefin (TPO) and polyolefin elastomer (POE)), compared to the most widely used Ethylene Vinyl Acetate (EVA) upon exposure to two different artificial ageing tests (with and without ultraviolet (UV) irradiation). Additive composition, optical and thermal properties and chemical structure (investigated by means of Thermal Desorption Gas Chromatography coupled to Mass Spectrometry, UV-Visible-Near Infrared spectroscopy, Differential Scanning Calorimetry, Thermogravimetric Analysis and Fourier Transform-Infrared spectroscopy, respectively) of the analyzed polymers were monitored throughout the exposure to artificial ageing tests. Relevant signs of photo-oxidation were detectable for TPO after the UV test, as well as a depletion of material’s stabilizers. Signs of degradation for EVA and POE were detected when the UV dose applied was equal to 200 kW h m^−2^. A novel approach is presented to derive information of oxidation induction time/dose from thermogravimetric measurements that correlate well with results obtained by using oxidation indices.

## 1. Introduction

Encapsulant materials are key components in photovoltaic (PV) modules because they need to provide and ensure several properties and functions. These materials provide optical coupling, give mechanical stability and support, protection to the electrical components from environmental factors and ensure electrical insulation for safety reasons [1].

Despite the requirements mentioned above, degradation of polymers used as encapsulants due to the effect of temperature, humidity and irradiation might lead to PV degradation modes such as delamination, corrosion and discoloration [2].

Material selection for encapsulants in PV has changed over time. If during the 1960s and the 1970s the prevailing encapsulant was based on polydimethylsiloxane (PDMS) because of its ultraviolet (UV) and thermal stability [3], nowadays Ethylene Vinyl Acetate (EVA) and Polyvinyl Butyral (PVB) are the market leaders for glass–backsheet and glass–glass modules, respectively [4]. The transition between the materials used as encapsulants has taken place mainly because of economic considerations as well as relatively proved reliability, rather than to outstanding EVA material properties. In fact, due to their chemical structure, PDMS based materials have less impact on mechanical stress on solar cells than EVA as well as higher bond dissociation energy. An energy of 108 kcal mol^−1^ is necessary to break Si−O bonds, whereas 83 kcal mol^−1^ is sufficient to break C−C bonds, typical of polyethylene based materials [3], such as EVA.

EVA is a copolymer characterized by ethylene and vinyl acetate moieties, the latter ranging between 28 wt.%. and 33 wt.%., and compounded with additives and stabilizers such as peroxides, crosslinking agents, antioxidants, UV absorbers and UV stabilizers [5].

Degradation mechanisms taking place due to the effect of temperature and irradiation have been extensively studied and reported in literature [1,6,7]. Norrish Type II and Norrish Type I are the main pathways that lead to the formation of acetic acid, polyenes and acetaldehyde, as well as other gases such as CO, CO_2_ and CH_4_ [1].

Despite relevant effort being made over the years to develop material properties and improve EVA encapsulant reliability, several degradation modes of PV modules can still be attributed to EVA degradation. Acetic acid, in particular, is a major cause of corrosion effects for metallization and cell connectors as well as delamination and potential induced degradation (PID) [8,9,10]. Negative material interaction between encapsulant, additives and electrical components of PV modules have been reported in literature as well [11,12,13,14].

Recently, an interest in new encapsulant materials has increased to overcome the issues due to the use of EVA and new formulations have been tested. In particular, Thermoplastic Polyolefines (TPO) and Polyolefin Elastomers (POE) have been introduced as alternatives to EVA as encapsulants for PV applications [8,15,16,17,18,19]. TPO and POE are polyethylene based materials, as well as EVA, but do not have vinyl acetate moieties. TPO and POE present in their structure as additional side groups, acrylates, acrylic acids and n-alkanes [8].

The main advantage of TPO and POE is that they cannot produce acetic acid during degradation because they do not have vinyl acetate moieties. POEs undergo chemical crosslinking, as well as EVA, therefore their formulation includes typically peroxides as crosslinking agents that react forming covalent bonds between the polymer chains. TPOs, instead, being thermoplastic materials, do not require peroxides or additional crosslinking agents to crosslink because they physically form hydrogen bonds upon the application of high temperatures. The materials mentioned above do not have particular drawbacks because they are similar to EVA in terms of costs and processing conditions. Additionally, the lack of vinyl acetate units limits the occurrence of corrosion and PID [8]. In a work published recently, Oreski et al. proved that module laminated with POE and TPO materials did not show signs of corrosion upon exposure to high temperatures and high humidity values [19].

Even though degradation of polyethylene based polymers has been largely studied and reported in literature [20,21,22], more in detail studies regarding formulations for PV applications are still in progress. Moreover, the influence of the materials formulation in terms of additives and their effects on encapsulant degradation over long term exposure is still an open question.

In this paper, two newly developed PV encapsulant materials (a TPO and a POE) have been subjected to artificial ageing tests and their performances have been compared to the most widely used encapsulant (EVA). The influences of the exposure to UV radiation, temperature and humidity have been studied and the effects on additive composition, chemical degradation and thermal stability are discussed. Bare polymer films, thermally pre-treated but not encapsulated within the typical PV module stack configuration have been the object of this study. The choice of using films has been made to better understand how environmental factors influence the degradation behavior of the polymer itself directly exposed and to have an insight of what could happen to the materials in case additional degradation modes, such as backsheet cracks and extensive delamination, might occur [23,24]. The work aims to make a step forward in understanding and comparing performances of newly developed material with respect to the state-of-the-art EVA by means of a comprehensive analysis that has not been performed before. Additionally, a new methodology that correlates oxidation indices measured via infrared spectroscopy and thermal stability indicators is presented.

## 2. Materials and Methods

Three types of polymer materials commercially available have been chosen in this study: an ultra-fast cure ethylene vinyl acetate (EVA, PHOTOCAP^®^ 15580P from Specialized Technology Resources), a thermoplastic polyolefin (TPO, Icosolar^®^ from Isovoltaic) and a polyolefin elastomer (POE, PO8110 from 3M). The materials mentioned above are all polyethylene based and used as encapsulants in photovoltaic applications. The samples were pre-treated in a vacuum laminator between two non-adhesive sheets at maximum temperature of 150 °C for a total duration of 20 min and were cut into stripes before being subjected to artificial ageing tests. The pre-treatment had the purpose of simulating the thermal treatment that the encapsulant experiences during the lamination process and to allow the crosslinking reaction to take place for the EVA and the POE encapsulant. If not crosslinked, the polymers would melt and flow at the exposure temperature. Main encapsulants characteristics are summarized in Table 1.

The samples were aged under damp heat (DH) test up to 3300 h in a climate chamber WLK 64–40 from Weiss Umwelttechnik GmbH. The temperature was set at 85 °C and relative humidity was set at 85%. Samples were withdrawn from the climate chamber after 1000 h, 1800 h, 2300 h and 3300 h of exposure and characterized according to the methods described in the following chapters.

To evaluate the effect of UV radiation, samples were aged in a UVTest™ Fluorescent UV/Condensation Weathering instrument from Atlas Material Testing Technology LLC. The test cycles were programmed according to the standard ISO 4892-3 Cycle 1 (Table 2), using fluorescent lamps with an irradiation peak at 340 nm and the maximum dose applied was 200 kW h m^−2^. Samples were withdrawn from the UV test after being exposed to a dose of 23 kW h m^−2^, 85 kW h m^−2^, 127 kW h m^−2^ and 200 kW h m^−2^ and characterized as described as follows.

### 2.1. Thermal Desorption Gas Chromatography Coupled to Mass Spectrometry (TD-GC/MS)

The Thermal Desorption Gas Chromatography coupled to Mass Spectrometry (TD-GC/MS) measurements were carried out to qualitatively analyze the additive composition of the commercial encapsulants before and after lamination, as well as after artificial ageing.

The thermal desorption phase took place in an EGA/Pyrolyzer-3030D from Frontier Laboratories Ltd. The sample, around 0.5 mg of material, was heated from 60 °C to 320 °C, with a heating rate of 20 °C min^−1^, and kept at 320 °C for three minutes; the maximum interface temperature was set at 300 °C. During this phase, the gaseous substances desorbed from the sample were collected and, when the process was completed, they were sent to the GC/MS (GC-MS QM2010 Ultra from Shimadzu) system and then analyzed. The column used was an Optima-5-Accent (length of 30 m, internal diameter of 0.25 mm, film thickness of 0.25 μm) and the carrier gas was helium.

The separation in the column was carried out using the following parameters:

- held for two minutes at 50 °C,
- from 50 C to 290 °C, held at 290 °C for six minutes, heating rate of 10 C min^−1^,
- ion source temperature and interface temperature were set at 300 °C,
- splitless mode.

The Mass Spectrometer was set in Scan Mode in the range from 50 *m/z* to 800 *m/z*, with ionization energy of 0.70 eV. The identification of the detected substances was performed by means of alignment with the National Institute of Standards and Technology (NIST) database.

### 2.2. UV-Visible-Near Infrared Spectroscopy (UV-Vis-NIR)

Hemispherical transmittance of the encapsulants before and over the exposure was recorded over the wavelength range between 250 nm and 2500 nm with a Lambda 950 UV-Visible-Near Infrared (UV-Vis-NIR) Spectrophotometer from PerkinElmer Inc.

### 2.3. Fourier Transform Infrared Spectroscopy (FT-IR)

Fourier Transform Infrared (FT-IR) spectroscopy measurements were performed using a Spectrum Two FT-IR Spectrometer from PerkinElmer Inc. in Attenuated Total Reflectance (ATR) mode using a MIRacle unit, equipped with a Zn/Se crystal with diamond tip. The spectra were measured in the interval 4000 cm^−1^ to 650 cm^−1^, averaging 16 scans with a resolution of 4 cm^−1^. The displayed spectra were normalized with respect to the intensity of the peak at 2850 cm^−1^, which was referred to methyl and methylene groups of polyethylene chains [25].

Oxidation Indices (OI) were evaluated to compare the overall oxidation state of the materials. The values were calculated as the ratio between the integral of the spectra from 1680 cm^−1^ to 1800 cm^−1^ (carbonyl region, related to oxidation products) and the reference band from 2760 cm^−1^ to 2875 cm^−1^ [18,26]. Results were normalized with respect to the initial value.

### 2.4. Differential Scanning Calorimetry (DSC)

Differential Scanning Calorimetry (DSC) was carried out using a DSC 6000 from PerkinElmer Inc. to measure thermograms of encapsulant materials before and after exposure. For each material, around 10 mg were placed in an aluminum pan and subjected to a first heating run from −70 °C to 180 °C, followed by a cooling run from 180 °C to −70 °C and a second heating run from −70 °C to 180 °C. During each step, heating (and cooling) rates were set to 10 K min^−1^ and a nitrogen flow of 50 mL min^−1^ was imposed.

Two heating steps were necessary to distinguish reversible changes due to physical processes, such as post crystallization, from irreversible chemical processes with effect on molecular structure. Melting enthalpies and temperatures were calculated by evaluating the area between the melting/crystallization peaks and the baseline. At least three measurements were performed for each sample at each ageing step. Crystallinity was calculated as the ratio between measured heat of fusion and the literature value for the 100% crystalline polyethylene (Δ*H*_m_^0^ = 293 J g^−1^) [27,28].

### 2.5. Thermogravimetric Analysis (TGA)

Thermogravimetric analysis (TGA) was performed by using a Thermogravimetric Analysis TGA/DSC 1 from Mettler Toledo GmbH. The weight loss of around 10 mg of encapsulant material was monitored while heating the sample in nitrogen atmosphere (50 mL min^−1^) from 25 to 600 °C, with a heating rate of 10 °C min^−1^. Temperature values at which the weight loss was equal to 5% with respect to the initial value (*T*_5_) were considered as an indicator for the beginning of the material’s decomposition process.

## 3. Results and Discussion

### 3.1. Thermal Desorption Gas Chromatography Coupled to Mass Spectrometry (TD-GC/MS)

TD-GC/MS measurements were performed to detect the stabilizers present in the encapsulants selected throughout their exposure to artificial ageing tests [29].

The analysis was performed qualitatively. In general, the area below the peaks in the chromatogram were related to concentration values, but the strong dependency of the peaks’ height on the amount of the material itself as well as on inhomogeneity of polymers did not allow, in this case, quantitative interpretations of results. Additionally, it is possible that not all the additives and stabilizers present in the encapsulants were detected. Nevertheless, the method is very useful to get insights regarding the main stabilization recipe for each material.

A butylated hydroxytoluene (BHT) was identified as an antioxidant for the EVA material as well as a UV absorber belonging to the benzophenone class (2-Hydroxy-4-(octyloxy)phenyl(phenyl)methanone. It was possible to detect both stabilizers after 3300 h of exposure in DH test. Additionally, a benzotriazole-type UV absorber (2-(2H-benzotriazol-2-yl)-4,6-bis(1,1-dimethylpropyl)phenol) was detected after 1000 h of DH exposure and until the end of the artificial ageing test [14,30]. Benzotriazole-type UV absorbers are soluble in water and it is possible that due to the high humidity values, a relevant fraction of the stabilizers migrated from the bulk to the surface of the material, hence facilitating thermal desorption processes and detection via TD-GC/MS. The combination of benzophenone and benzotriazole based stabilizers has been proven to give a positive effect on the ageing behavior of polyolefin based materials [31]. After the exposure to UV test, the BHT antioxidant was detected up to 85 kW h m^−2^, whereas the benzophenone based UV absorber was detected up to 200 kW h m^−2^ and no additional benzotriazole-type UV absorber could be identified.

A fragment of BHT antioxidant [32] as well as an additional sterically hindered phenolic antioxidant (Octadecyl 3-(3,5-di-tert-butyl-4-hydroxyphenyl)propionate, also known as Antioxidant 1076) were detected in unexposed TPO encapsulant. At the end of the DH test it was still possible to detect the BHT antioxidant, but it was no longer possible to detect the hindered phenolic antioxidant. As for EVA, it was possible to detect after 1000 h of the DH test and up to 3300 h, a benzotriazole-type UV absorber. Extensive degradation of this encapsulant could be assessed after the exposure to UV radiation. A dose of 85 kW h m^−2^ was sufficient to deplete all the stabilizers present in the bare material, as it was no longer possible to detect any stabilizer. Details of resulting degradation processes will be given in the next sections. Additionally, at the end of the UV exposure test (applied dose of 200 kW h m^−2^), only degradation products of the encapsulant itself were detectable.

The analysis carried out on the POE sample before the exposure to artificial ageing tests showed the presence of a BHT antioxidant, which was no longer detected after 1000 h of DH test. A benzotriazole-type UV absorber was instead detected after 1000 h of DH exposure. During UV test, the BHT was no longer detected, while traces of the hindered phenolic antioxidant were detected up to a dose of 200 kW h m^−2^.

A summary of the detected stabilizers over the exposure to the artificial ageing tests is shown in Table 3. Additional details regarding the results are available online as Supplementary Material (Figures S1–S4).

### 3.2. UV-Visible-Near Infrared Spectroscopy (UV-Vis-NIR)

**Figure 1:**
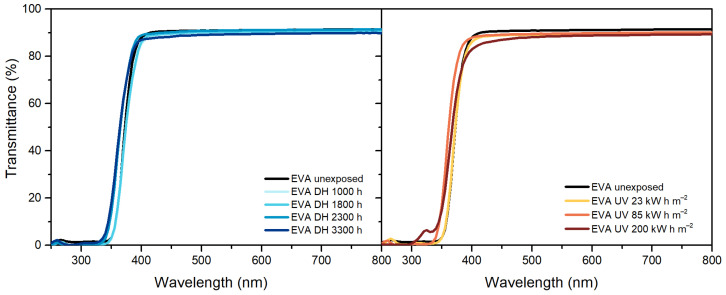
Ultraviolet-Visible (UV-Vis) spectra of ethylene vinyl acetate (EVA) unexposed, exposed to damp heat (DH) (**left**) and ultraviolet (UV) test (**right**).

Maximum transmittance values of EVA, Figure 1, decreased over the exposure in both the DH and UV tests from (from 91% to 89.9% and from 91% to 89.3%, respectively). After exposure to a dose of 200 kW h m^−2^ a decrease in transmittance values in the blue range of the spectra (380 nm–500 nm) was connected to a yellowing of the material. A slight decrease of the UV cut-off values was observable as well as an increase of the transmittance value in the region between 250 and 350 nm, which might be correlated to the consumption of the UV absorbers. Additionally, the peak present at around 260 nm, possibly associated to presence of the BHT antioxidant, disappeared after a dose of 200 kW h m^−2^ and this result is in good accordance with the results from qualitative additive analysis.

**Figure 2:**
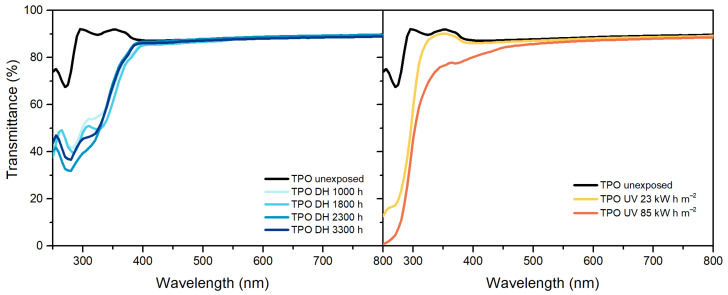
UV-Vis spectra of thermoplastic polyolefin (TPO) unexposed, exposed to DH (**left**) and UV test (**right**).

In the case of TPO, Figure 2, results show transmittance values higher than 90% below 390 nm. After the exposure to DH test, it is possible to observe a decrease of transmittance value below 390 nm, but the maximum value of transmittance in the visible range remains the same. The samples exposed to 127 kW h m^−2^ and to 200 kW h m^−2^ showed very strong signs of embrittlement and it was not possible to perform the measurements on these materials. Nevertheless, already after a dose of 85 kW h m^−2^ the material shows a decrease of transmittance below 390 nm as well as above. The decrease between 380 nm and 500 nm can be attributed to chromophore formation that results in material discoloration [33].

**Figure 3:**
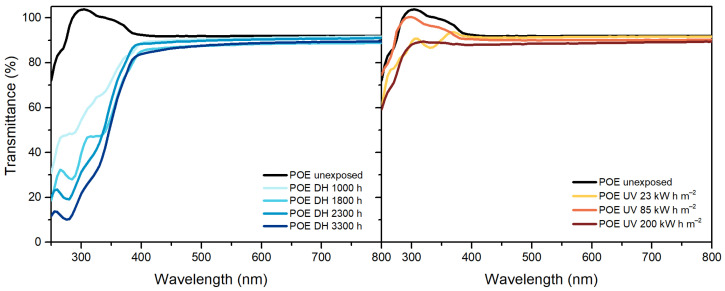
UV-Vis spectra of polyolefin elastomers (POE) unexposed, exposed to DH (**left**) and UV test (**right**).

POE, similarly to TPO, showed a transmittance value higher than 90% below 390 nm, which decreased upon UV exposure (Figure 3). The maximum transmittance in the visible range decreased from 92% to 90% and finally to 88%. During DH exposure, instead, transmittance values decreased in the UV range as well as in the visible range below 500 nm indicating that the material’s yellowing was due to chromophore formation [33]. Formation of chromophores as well as migration of additives might have caused also the changes in transmittance below 390 nm, observable for POE and TPO [19,33].

### 3.3. Fourier Transform Infrared Spectroscopy (FT-IR)

**Figure 4:**
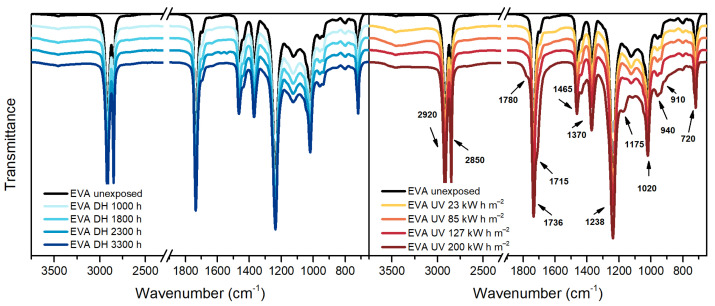
Fourier Transform Infrared in Attenuated total reflectance mode (FT-IR ATR) Spectroscopy measurements of EVA samples before and after exposure to DH (**left**) and UV test (**right**).

Analysis of degradation behavior of EVA can be performed by means of FT-IR ATR Spectroscopy measurements, Figure 4. The bands that can be seen at 2920 cm^−1^, 2850 cm^−1^, 1465 cm^−1^ and 1370 cm^−1^ can be assigned to stretching and deformation vibrations of methylene and ethylene groups, whereas the band at 720 cm^−1^ can be assigned to rocking vibrations of ethylene groups. The bands mentioned above are all characteristics of polyethylene [34].

Vinyl acetate moieties can be identified from the bands at 1736 cm^−1^ (corresponding to C=O stretching vibrations), 1238 cm^−1^ and 1020 cm^−1^, which can be assigned to C-O-C stretching vibrations [35].

The EVA spectra after 3300 h of exposure in the climate chamber does not show presence of new peaks. Photo-thermal degradation processes, instead, took place during UV exposure and it was possible to observe the presence of new peaks due to the formation of new functional groups. The typical thermal degradation pathway for EVA involves the production of acetic acid followed by main chain decomposition processes. Polyethylene chains, as result of thermal degradation, form hydroperoxide groups that can further react and form various carbonyl groups. The formation of the shoulder at 1780 cm^−1^ can be attributed to the formation of γ-lactone due to back-biting process of vinyl acetate moieties [35]. The broadening of the shoulder at 1715 cm^−1^ as well as the band at 1175 cm^−1^ are associated to ketones. Those species might be produced by means of acetaldehyde evolution or breakdown of hydroperoxides. The characteristic bands of EVA as well as the appearance of new bands with their assignment are summarized in Table 4.

**Figure 5:**
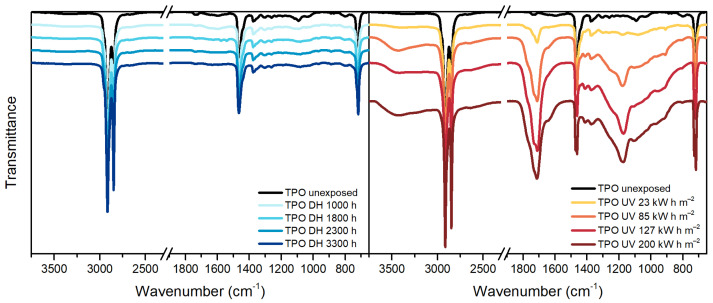
FT-IR ATR Spectroscopy measurements of TPO samples before and after exposure to DH (**left**) and UV test (**right**).

TPO, Figure 5, is a polyethylene based encapsulant and, therefore, it shows in the IR spectra the typical peaks of polyethylene at 2920 cm^−1^, 2850 cm^−1^, 1465 cm^−1^, 1370 cm^−1^ and 720 cm^−1^. Additionally, the unexposed material shows the presence of a band around 1600 cm^−1^ that might be attributed to the C=C aromatic bonds of the hindered phenolic antioxidant [36], detected by means of TD-GC/MS as well.

After the exposure in DH test, the spectrum does not show relevant changes. The dose of 85 kW h m^−2^ during the UV test was, instead, already sufficient to cause damages to the material. The main differences with the unexposed material are noticeable in the ranges 800 cm^−1^–1400 cm^−1^, 1680 cm^−1^–1800 cm^−1^ and 3100 cm^−1^–3700 cm^−1^ corresponding to unsaturation, carbonyl and hydroxyl regions, respectively. The material exposed to 200 kW h m^−2^ of UV dose showed a progression of the damage and extensive effects.

According to the work published by Yagoubi et al. [26], macro-radicals are formed when macromolecules absorb energy from UV light. Subsequently, macro-radicals can react with the surrounding oxygen molecules and light can catalyze the formation of substances that eventually react with the polymer chain giving hydroperoxides. These latter can further decompose and produce ketones, aldehydes, carboxylic acids and esters through different routes.

Peaks similar to TPO can be identified in the unexposed POE sample. Typical peaks of polyethylene-based materials can be identified and they are summarized in Table 5.

**Figure 6:**
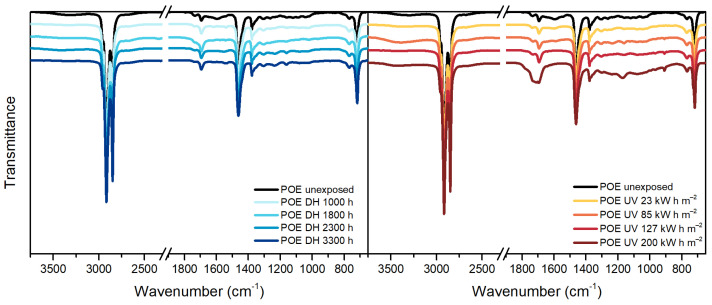
FT-IR ATR Spectroscopy measurements of POE samples before and after exposure to DH (**left**) and UV test (**right**).

During DH exposure no relevant changes were detected, see Figure 6, and slight differences were seen after UV exposure. In particular, it is possible to notice the formation of a new peak at 909 cm^−1^ corresponding to vinyl group, as well as in the carbonyl region, between 1670 cm^−1^ and 1800 cm^−1^, and in the hydroperoxide region, between 3100 cm^−1^ and 3700 cm^−1^. The presence of a peak around 1715 cm^−1^ and 1175 cm^−1^ indicates the formation of ketones. The ageing mechanism seems to be similar to the one taking place in the TPO encapsulant (but less pronounced), as expected since the two materials are similar from the chemical point of view.

**Figure 7:**
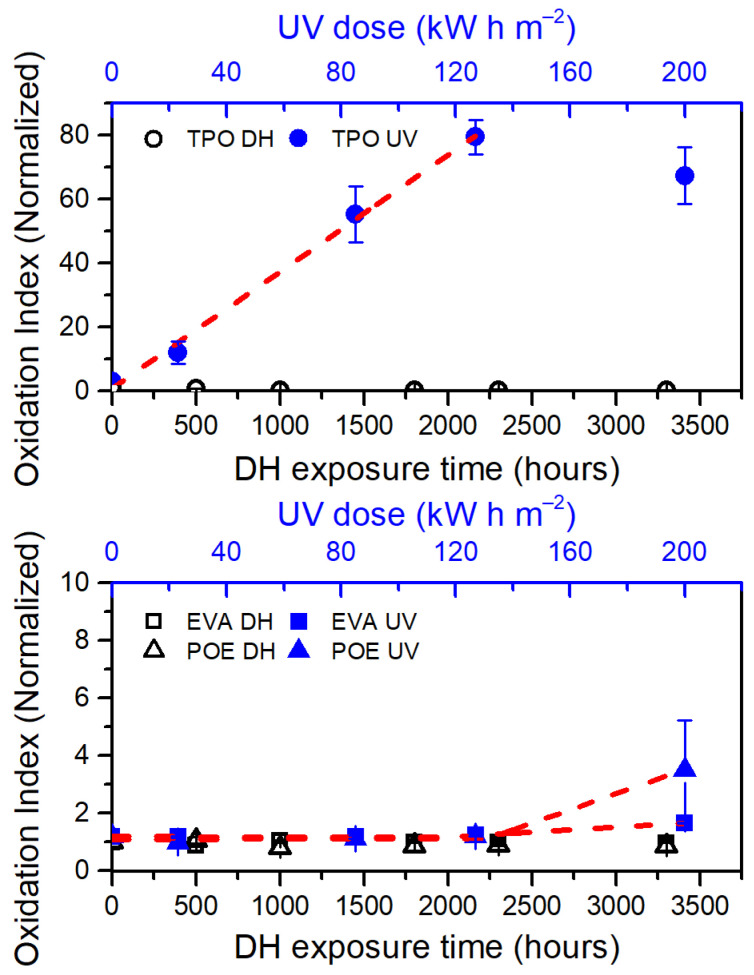
Oxidation Index (OI) versus exposure time (DH test, open symbols in black color) and dose (UV test, full symbols in blue color). Red dashed lines are fitting functions applied to the data to extrapolate the oxidation induction time/dose. TPO (**top** chart), EVA and POE (**bottom** chart).

Oxidation Indices (OI) have been evaluated to compare the overall oxidation state of the materials. The values have been calculated as the ratio between the integral of the spectra from 1680 cm^−1^ to 1800 cm^−1^ (carbonyl region, related to oxidation products) and the reference band from 2760 cm^−1^ to 2875 cm^−1^. Results have been normalized with respect to the initial value and are shown in Figure 7.

The samples exposed to DH test did not show any substantial change, whereas each encapsulant shows signs of oxidation after UV exposure. An increase of almost 1.5 times compared to the initial values can be observed for the EVA material when the UV dose applied is 200 kW h m^−2^. By applying a linear piecewise fitting function to the data, the dose from which the material begins to show oxidation is extrapolated. To evaluate the accordance of the fitting function to the data, R^2^ values have been taken into account. In the case of EVA, the fitting function shows an R^2^ value of 0.997 and a dose of about 113 kW h m^−2^ is sufficient to initiate oxidation. The final value of OI is about 3.4 times higher than the initial one for POE encapsulant and a dose of about 131 kW h m^−2^ (R^2^=0.984) is necessary to initiate degradation.

TPO is the material that shows the most severe degradation. Considering only the first four steps of the artificial ageing tests, a linear fit can be applied to the data and an OI increasing rate of 0.0293 m^2^ kW^−1^ h^−1^ is obtained, with an R^2^ value of 0.994. The OI value calculated at 127 kW h m^−2^ is almost 80 times higher than the initial value. The OI value at the final stage of the UV test is considered as outlier in this analysis because of the very strong physical degradation, associated to chemical processes.

The occurrence of the oxidation processes correlates well with consumption of antioxidants, for all the materials, as detected by means of TD-GCMS.

### 3.4. Differential Scanning Calorimetry (DSC)

Samples aged under different accelerated ageing procedures showed significant changes in thermal behavior. In general, thermal properties can be influenced by both physical and chemical ageing processes. Chemical processes such as chain scission that might occur during UV ageing due to photo-oxidation are responsible for chemo-crystallization, namely secondary crystallization. The effects of chemical ageing are irreversible and can be detected by observing changes in melting enthalpy and temperature in the second heating curve. The first heating curve might show the same signs of degradation, but they are the results of a combination of chemical and physical ageing. Physical processes have similar effects to the ones due to annealing at high temperatures, changes in melting temperature and enthalpy might occur and are visible in the first heating curve. No reversible effects of physical ageing can be seen in the second heating curve [27,37,38].

**Figure 8:**
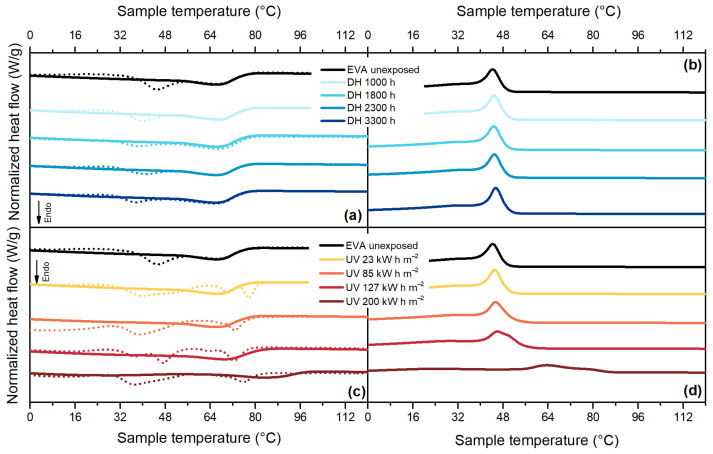
Differential scanning calorimetry (DSC) thermograms for EVA. First heating curves (dashed lines) and second heating curves (full lines) from samples exposed to DH test (**a**). Cooling curves for samples exposed to DH test (**b**). First heating curves (dashed lines) and second heating curves (full lines) from samples exposed to UV test (**c**). Cooling curves for samples exposed to UV test (**d**).

For EVA, Figure 8a, it is possible to notice that there is a change in its thermal behavior in the first heating cycle. The main melting peak of EVA, with maximum at 66 °C, does not change its position, even after 3300 h of DH test. The secondary melting peak, instead, shifts towards lower temperature values from 45 °C to 37 °C indicating a physical change in the crystal population of the material, not associated with chemical degradation. The presence of vinyl acetate moieties in EVA induced a different ability of the material to crystallize. Ethylene segments in vinyl acetate moieties have the tendency to form smaller and less perfect crystals, which melt at lower temperatures compared to the polyethylene units [18,39,40].

During the UV test, Figure 8c, a combination of effects from physical and chemical ageing can be observed. On one side, the first heating run reveals the presence of a shoulder below the main melting temperature and a shift of this latter towards higher values (from 66 °C to 83 °C). This is a typical effect that can occur when the annealing (ageing) temperature lies within the melting region. During the exposure in the test chamber at 60 °C, smaller crystals with thinner lamellae can melt and crystallize again to form more perfect crystals with thicker lamellae, which melt at higher temperatures [27,37]. Additionally, in the last two steps of the UV test, it is possible to notice a shift of the melting temperature visible in the second heating run of the DSC measurement from 66 °C to 68 °C and finally to 86 °C, which is an indicator for the occurrence of deacetylation reaction [18,39]. Signs of chemo-crystallization are noticeable also when looking at the cooling curve of the thermogram (Figure 8d) because the crystallization peak is shifted to much higher temperatures and the temperature range in which the crystallization process takes place is enlarged.

**Figure 9:**
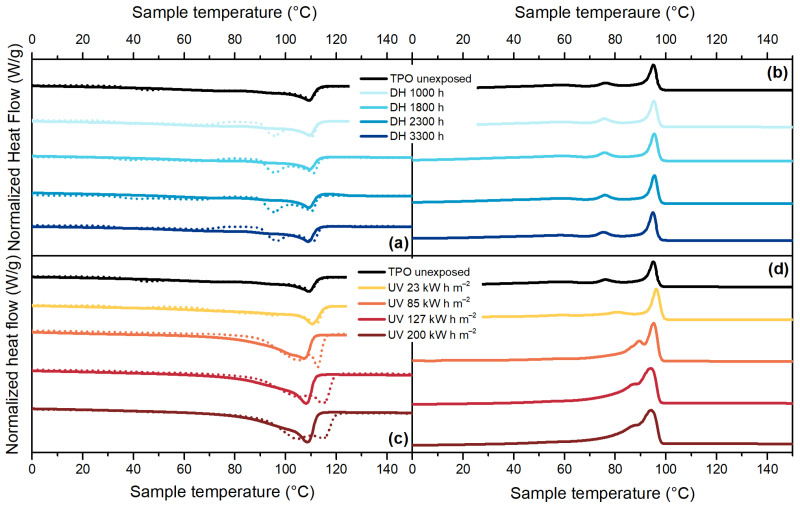
DSC thermograms for TPO. First heating curves (dashed lines) and second heating curves (full lines) from samples exposed to DH test (**a**). Cooling curves for samples exposed to DH test (**b**). First heating curves (dashed lines) and second heating curves (full lines) from samples exposed to UV test (**c**). Cooling curves for samples exposed to UV test (**d**).

TPO material, Figure 9a, showed a main melting peak at around 109 °C as well as a shoulder at about 94 °C, which was no longer visible during the second heating run. During DH test exposure, no significant changes in crystallinity and melting temperature were observed. Only a rearrangement of the crystal population can be seen in the first heat run, similarly to EVA. During UV exposure, instead, the crystallinity significantly increased from 30% (unexposed sample) to 50% (sample exposed to 200 kW h m^−2^). Additionally, the shoulder’s temperature increases from 94 °C to 105 °C, whereas the main melting peak’s temperature shifts from 109 °C to almost 115 °C, as can be seen in Figure 9c. The shift of the main melting peak is not visible when looking at the thermogram of the second heating run. During the cooling cycle, Figure 9d, there was an increase of the crystallization peak’s temperature from 95 °C (unexposed material) up to 98 °C (sample exposed to 85 kW h m^−2^) followed by a decrease down to 94 °C for the sample exposed to the maximum dose (200 kW h m^−2^). Additionally, the peak visible at around 76 °C was shifted to about 86 °C.

**Figure 10:**
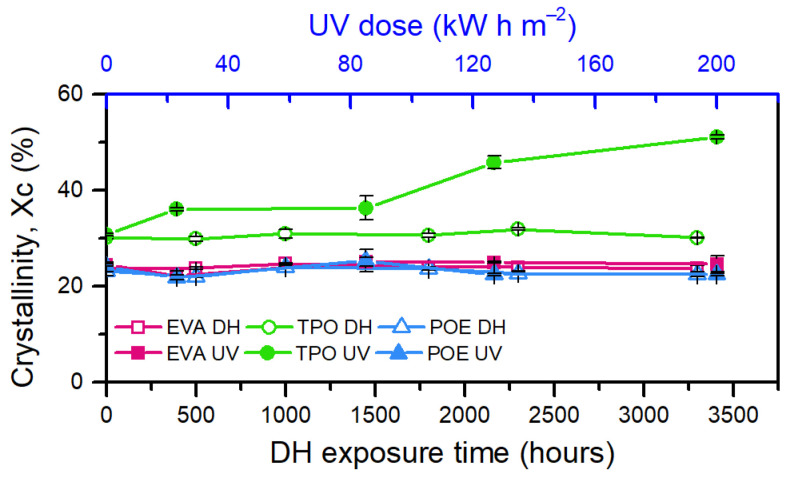
Evolution of crystallinity over UV and DH exposure of the encapsulants (EVA, TPO, and POE).

In this case, there was a competitive effect between changes in thermal behavior due to chain scission processes and crosslinking phenomena. These phenomena take place simultaneously when photo-oxidation reactions take place, as in the case of UV exposure, and both are responsible for severe embrittlement of the material [41,42,43]. Due to chain scission there was a formation of smaller chains that work as nucleating agents. A larger number of nuclei was present, and this led to an increase in crystallinity, Figure 10, as well as an increase in crystallization temperature. Additionally, the presence of these smaller nuclei is shown by the tail of the crystallization peak that is shifted towards lower temperature [27].

**Figure 11:**
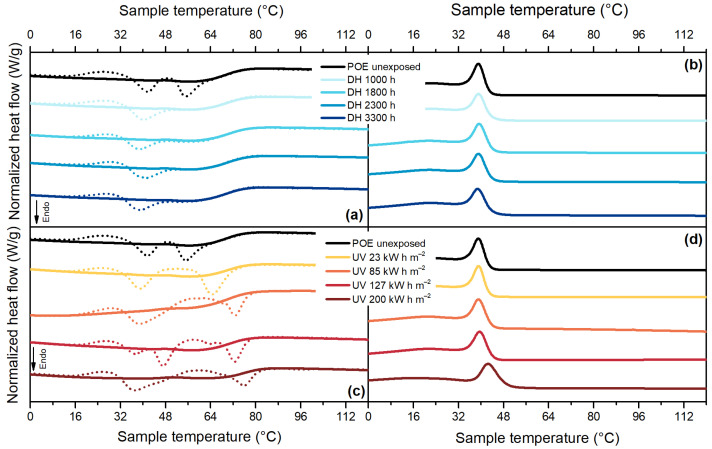
DSC thermograms for POE. First heating curves (dashed lines) and second heating curves (full lines) from samples exposed to DH test (**a**). Cooling curves for samples exposed to DH test (**b**). First heating curves (dashed lines) and second heating curves (full lines) from samples exposed to UV test (**c**). Cooling curves for samples aged under UV test (**d**).

The thermogram of POE, Figure 11a, shows a main melting peak at about 55 °C for the unexposed material and a secondary melting peak at about 41 °C. In the second heating run, the secondary melting peak disappears, thus showing a difference in the crystals’ population.

After DH exposure, it is possible to notice that the secondary melting peak slightly shifts to lower temperatures, from 41 °C to 39 °C, whereas the main melting peak appears more similar to a shoulder at around 56 °C. After the exposure to UV radiation significant effects of physical ageing can be observed, Figure 11c. For POE encapsulant, as well as for EVA, the temperature of the ageing test (65 °C during the light cycle and 50 °C during the dark cycle) played an important role in influencing the crystal population present in the material because it was within the melting interval. Two melting peaks were detected at 39 °C and at 76 °C (after a dose of 200 kW h m^−2^). The second heating run showed signs of chemical degradation due possibly to chain scission because the melting temperature increased from 55 °C to 62 °C. The occurrence of chain scission was supported by the increase of crystallization temperature from 39 °C to almost 43 °C and by the shift of the tail of the crystallization process towards lower temperatures, Figure 11d.

### 3.5. Thermogravimetric Analysis (TGA)

TGA measurements on the encapsulants before and after the exposure to the artificial ageing tests were carried out to monitor the evolution of the thermal stability of the materials.

**Figure 12:**
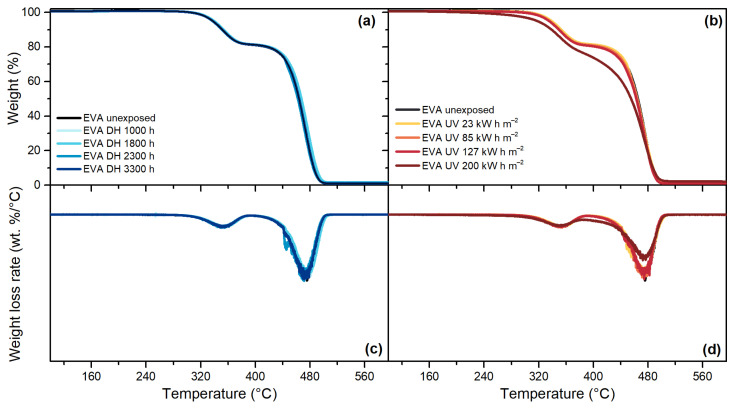
Residual weight over temperature during Thermogravimetric Analysis (TGA) measurements of EVA after the exposure to DH test (**a**). Residual weight over temperature during TGA measurements of EVA after the exposure to UV test (**b**). Weight loss rate over temperature of EVA samples exposed to DH test (**c**). Weight loss rate over temperature of EVA samples exposed to DH test (**d**).

For the EVA material, Figure 12, the decomposition process took place in two steps: the first step was typical of the cleavage of vinyl acetate moieties, whereas the second step corresponded to the decomposition of the main polyethylene chains. As can be seen from the first derivative of the thermogram, the maximum weight loss rate during the deacetylation and polyethylene decomposition reaction was detectable at around 350 °C and 474 °C, respectively.

**Figure 13:**
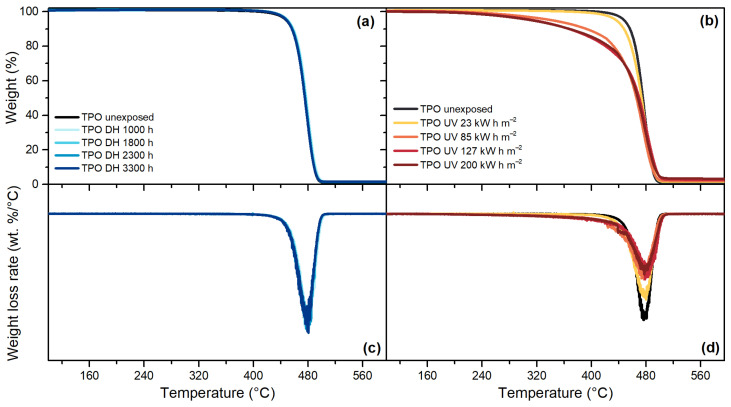
Residual weight over temperature during TGA measurements of TPO after the exposure to DH test (**a**). Residual weight over temperature during TGA measurements of TPO after the exposure to UV test (**b**). Weight loss rate over temperature of TPO samples exposed to DH test (**c**). Weight loss rate over temperature of TPO samples exposed to DH test (**d**).

**Figure 14:**
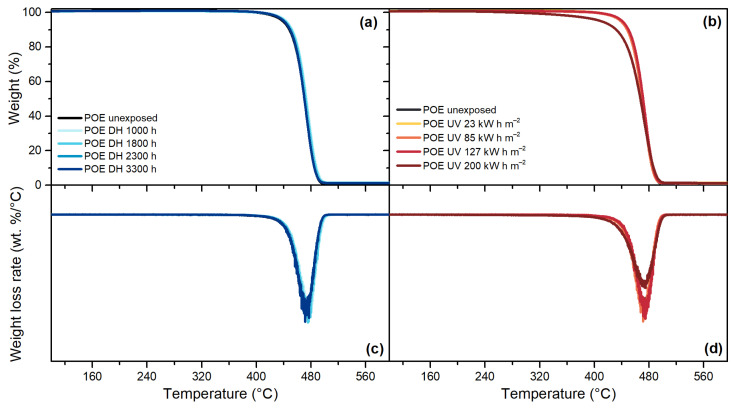
Residual weight over temperature during TGA measurements of POE after the exposure to DH test (**a**). Residual weight over temperature during TGA measurements of POE after the exposure to UV test (**b**). Weight loss rate over temperature of POE samples exposed to DH test (**c**). Weight loss rate over temperature of POE samples exposed to DH test (**d**).

TPO, Figure 13, and POE, Figure 14, are characterized by a one-step decomposition process, with a maximum weight loss rate at around 478 °C and 474 °C, respectively. The exposure to DH test did not show significant differences for any of the encapsulants, as can be seen from the good overlapping of the thermograms of the unexposed and the materials exposed to DH test after 3300 h. Signs of degradation were detectable after the UV test. For TPO, the thermogram of the material exposed to a dose of 85 kW h m^−2^ shows already a deviation from the unexposed material and the deviation is visible also for materials exposed to higher doses. The same trend can be seen when looking at the weight loss rate curve; the intensity of the peak, indeed, is halved after 85 kW h m^−2^ with respect to the unexposed encapsulant. For POE, the thermograms overlap to the unexposed material except that for the materials exposed to 200 kW h m^−2^, evident also from the weight loss rate curve that shows a lower intensity of the peak.

**Figure 15:**
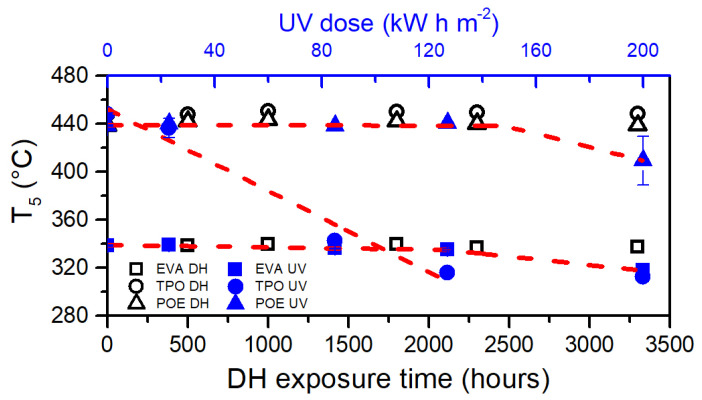
Temperature corresponding at 95% residual weight (T_5_) versus exposure time (DH test, open symbols in black color) and dose (UV test, full symbols in blue color). Red dashed lines are linear fittings applied to the data to extrapolate the oxidation induction time/dose.

Figure 15 summarizes the temperature at which each material reached a residual weight of 95%, defined as T_5_. Photo-degradation processes that took place during UV test significantly lowered the thermal stability of TPO. In fact, the decomposition process began at much lower temperatures compared to the unexposed material. A dose of 85 kW h m^−2^ was sufficient to cause a 5% weight drop at around 343 °C instead of 448 °C (106 °C of difference). Considering the first four steps of the UV test and applying a linear fit to the data a value of R^2^ = 0.96 was obtained, thus indicating a linear decrease of the thermal stability upon the UV dose up to 127 kW h m^−2^. The value measured for TPO exposed to 200 kW h m^−2^, also in this analysis, results as an outlier compared to the other data. The results mentioned above are in good accordance to what has been shown with the analysis of the OI and with the evolution of the thermal properties measured with DSC. It can be derived that UV doses lower that 130 kW h m^−2^ are sufficient to severely degrade a bare TPO encapsulant.

The dose of 200 kW h m^−2^ is sufficient to decrease the thermal stability of EVA and POE because the aged material reaches T_5_ at lower temperatures compared to the unexposed materials of about 21 °C and 29 °C, respectively. A linear piecewise fitting can be applied to the data of EVA and POE to extrapolate the UV dose necessary to cause significant decrease of the thermal stability. Doses of 127 kW h m^−2^ (R^2^ = 0.994) and of 148 kW h m^−2^ (R^2^ = 0.982) for EVA and POE, respectively, can be extrapolated.

**Figure 16:**
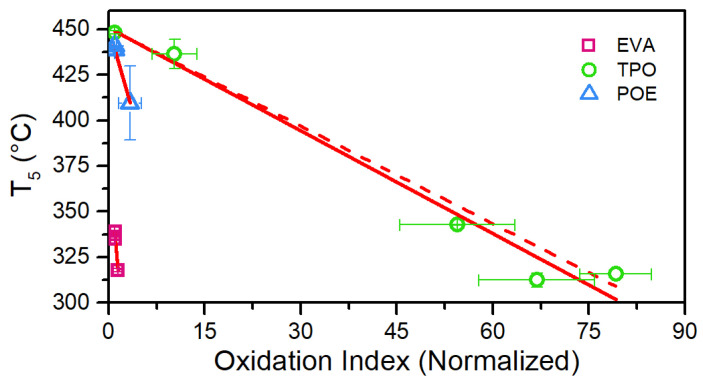
T_5_ values vs. Normalized Oxidation Index. Solid lines correspond to the linear fitting applied to the datasets for EVA and POE. For TPO, the solid line corresponds to the fitting applied to the whole dataset, whereas the dashed line corresponds to the fitting applied to four points over five.

Comparing the evolution of oxidation indices and T_5_ values over the UV dose, the results show a very good agreement (Figure 16). A linear fitting has been applied to each dataset and R^2^ values of 0.968, 0.980 and 0.983 have been determined for EVA, TPO and POE, respectively. In the case of TPO, the linear fitting is applied neglecting the value corresponding to an OI equal to 67, because it corresponds to the severely degraded material. If the whole dataset is considered the R^2^ value obtained for the fitting is equal to 0.967.

T_5_ values might be used as indicators to detect oxidation processes taking place in materials subject to ageing. The dose at which the temperature values of the exposed materials deviate from the unexposed ones correlates well with the increase of oxidation index evaluated using FT-IR spectroscopy measurements and with depletion of the antioxidants. To the best knowledge of the authors, the analysis mentioned above is presented for the first time in this work.

To summarize, as can be seen in Table 6, all the encapsulants showed a good behavior upon DH test and no significant material changes could be detected. The direct exposure to UV radiation, instead, provoked significant damages especially to TPO. Material stabilizers have been rapidly consumed and the chemical structure, thermal stability and morphology experienced severe changes. POE is the material that showed most similarities to EVA with the important advantage that this material does not present the formation of acetic acid upon ageing. Therefore, it seems to be a very promising material for PV applications.

## 4. Conclusions

The aim of the work was to compare the stability and degradation behavior characteristics of two different types of emerging encapsulant materials for PV modules, a TPO and a POE, to the most widely used EVA encapsulant under the influence of two different artificial ageing tests. The choice of testing bare films allows a better understanding of what happens to the materials when they are directly exposed, a circumstance that might occur or partially occur in concomitance of degradation modes such as backsheet cracks and extensive delamination. Additionally, testing bare materials highlights even more the importance of PV module components, such as glazing and insulating adhesives that prevent polymers from degradation.

Results showed that the additives and stabilizers are qualitatively detectable by means of TD-GC/MS for both exposed and unexposed samples. In particular, additives detected in the unexposed materials were generally present at the end of the exposure to the DH test. UV testing, instead, is much more severe and causes depletion of stabilizers and subsequent polymer degradation. Analysis of surface chemistry by means of FT-IR Spectroscopy confirmed that photo-oxidation reactions take place during the exposure to UV radiation and that the effects are particularly pronounced for TPO, which seems to be the less stabilized polymer compared to the others. The results regarding the evolution of thermal properties with exposure are in good agreement with the typical behavior of polyethylene-based polymers experiencing photo-oxidative degradation. Summarizing, EVA and POE encapsulants showed very good stability upon DH exposure, whereas they showed initial signs of photo-oxidation properties upon a dose of 200 kW h m^−2^. TPO, instead, showed good behavior upon DH exposure, but poor performances upon exposure to UV.

UV test reproduces in a more reliable way what actually happens in the field during outdoor exposure, although the damages that the polymers have faced are over estimated and more severe than the damages that the materials might have experienced in the usual PV module stack configuration. Nevertheless, findings from this experimental setup might be transferred to encapsulated samples with a different microclimate. Especially the oxidation induction time analysis carried out using the evolution of Carbonyl Index as well as evaluation of T_5_ values from TGA thermograms might be extended to different fields of polymer degradation studies.

Further work might be focused on correlating degradation mechanisms of the bare materials to the encapsulated ones, simulating moisture and oxygen ingress as well as backsheet cracks and delamination. Additionally, a better stabilization might be considered to improve weather resistance of materials that showed stronger degradation.

## Figures and Tables

**Table 1:** Encapsulants characteristics.

Encapsulant	Thickness (µm)	Chemical Crosslinking	Acetic Acid
EVA	450	Yes, with peroxides	Yes
TPO	500	No	No
POE	550	Yes, with peroxides	No

Ethylene vinyl acetate (EVA), thermoplastic polyolefin (TPO), polyolefin elastomer (POE).

**Table 2:** UV test cycles parameters.

Function	Irradiation (W m^−2^ nm^−1^)	Black Panel Temperature (°C)	Time (Hours:Minutes)
UV light	0.76	60	8:00
Condensation	n/a	50	4:00

Ultraviolet (UV).

**Table 3:** Summary of thermal desorption gas chromatography coupled to mass spectrometry (TD-GCMS) measurements on the encapsulants exposed to damp heat (DH) and ultraviolet (UV) tests.

**EVA**					
**Stabilizer**	**Unexposed**	**DH Ageing Time** **3300 h**	**UV Dose** **85 kW h m^−2^**	**UV Dose** **127 kW h m^−2^**	**UV Dose** **200 kW h m^−2^**
Antioxidant butylated hydroxytoluene (BHT)	✓	✓	✓	✓	n. d.
UV absorber (benzophenone)	✓	✓	✓	✓	✓
UV absorber (benzotriazole)	n. d.	✓	n. d.	n. d.	n. d.
**TPO**					
**Stabilizer**	**Unexposed**	**DH ageing time** **3300 h**	**UV dose** **85 kW h m^−2^**	**UV dose** **127 kW h m^−2^**	**UV dose** **200 kW h m^−2^**
Antioxidant (Antioxidant 1076)	✓	fragment	n. d.	n. d.	n. d.
UV absorber (benzotriazole)	n. d.	✓	n. d.	n. d.	n. d.
**POE**					
**Stabilizer**	**Unexposed**	**DH ageing time** **3300 h**	**UV dose** **85 kW h m^−2^**	**UV dose** **127 kW h m^−2^**	**UV dose** **200 kW h m^−2^**
Antioxidant (BHT)	✓	fragment	n. d.	n. d.	n. d.
UV absorber (benzotriazole)	n. d.	✓	n. d.	n. d.	n. d.
Antioxidant (Antioxidant 1076)	n. d.	n. d.	traces	traces	traces

n. d.: not detected.

**Table 4:** Assignment of FT-IR bands in EVA spectra.

Wavenumber [cm^−1^]	Assignment
2920	Asymmetric stretching vibration of CH_2_
2850	Symmetric deformation vibration of CH_2_
1780	C=O stretching vibration of γ-lactones
1715/1175	C=O stretching vibration of ketones
1736	C=O stretching vibration
1465	Asymmetric deformation vibration of CH_2_
1370	Symmetric deformation of CH_3_
1238	C-O-C stretching vibration
1020	C-O-C stretching vibration
960–940	CH out-of-plane deformation vibration of vinyl ether
910	CH out-of-plane deformation vibration of vinyl
720	CH_2_ skeleton rocking vibration

**Table 5:** Assignment of Fourier Transform Infrared (FT-IR) bands in POE spectra.

Wavenumber [cm^−1^]	Assignment
2920	Asymmetric stretching vibration of CH_2_
2850	Symmetric stretching vibration of CH_2_
1800–1680	C=O stretching vibration
1715/1175	C=O stretching vibration of ketones
1465	Asymmetric deformation vibration of CH_2_
1370	Symmetric deformation of CH_3_
909	CH out-of-plane deformation vibration of vinyl
720 (doublet)	CH_2_ skeleton rocking vibration

**Table 6:** Summary of encapsulants characteristics and performances observed in this study.

	EVA	POE	TPO
**Chemical crosslinking**	Yes	Yes	No
**Acetic acid**	Yes	No	No
**General DH stability after 3300 h**	Very good	Very good, transmittance decreases in UV range	Very good, transmittance decreases in UV range
**Presence of stabilizers upon UV exposure**	Yes	Partial	No
**Optical properties upon UV exposure**	Slight transmittance decrease	Slight transmittance decrease	Not measurable
**Chemical oxidation upon UV exposure**	Initial stage	Initial stage	Severe
**Crystallinity changes upon UV exposure**	Not relevant	Not relevant	Yes
**Thermal stability upon UV exposure**	Decreased	Decreased	Very much decreased

## Data Availability

Data presented in this study are available upon request from the corresponding author.

## Supplementary Materials

The following are available online at https://www.mdpi.com/2073-4360/13/2/271/s1, Figure S1: Chromatograms of EVA samples exposed to DH test (left) and UV test (right); Figure S2: Chromatograms of TPO samples exposed to DH test (left) and UV test (right); Figure S3: Chromatograms of POE samples exposed to DH test (left) and UV test (right); Figure S4: Mass spectra of the additives detected by means of TD-GC/MS.
