# Supplementary material for: Comparison of Degradation Behavior of Newly Developed Encapsulation Materials for Photovoltaic Applications under Different Artificial Ageing Tests
Source: Polymers (Basel). 2021 Jan 15;13(2):271. doi: 10.3390/polym13020271 (PMC7830908; doi:10.3390/polym13020271)
Supplement: Supplementary file 1 [file polymers-13-00271-s001.pdf]

polymers

MDPI

Supplementary Materials: Comparison of Degradation Behavior of Newly Developed Encapsulation Materials for Photovoltaic Applications Under Different Artificial Ageing Tests

Chiara Barretta, Gernot Oreski, Sonja Feldbacher, Katharina Resch-Fauster and Roberto Pantani

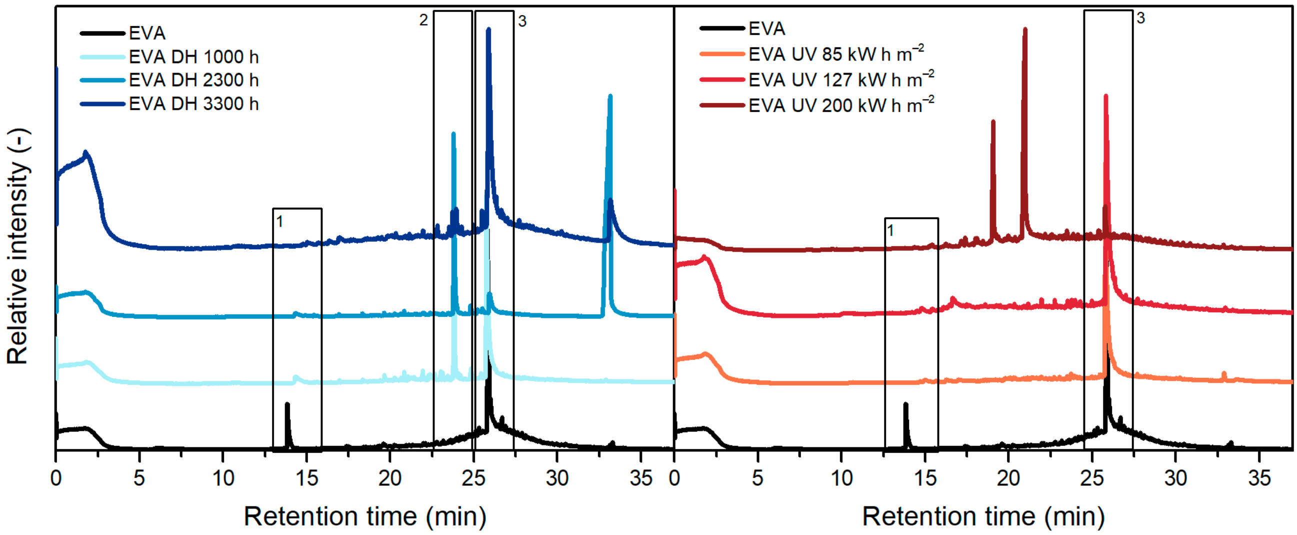

Figure S1. Chromatograms of EVA samples exposed to DH test (left) and UV test (right).

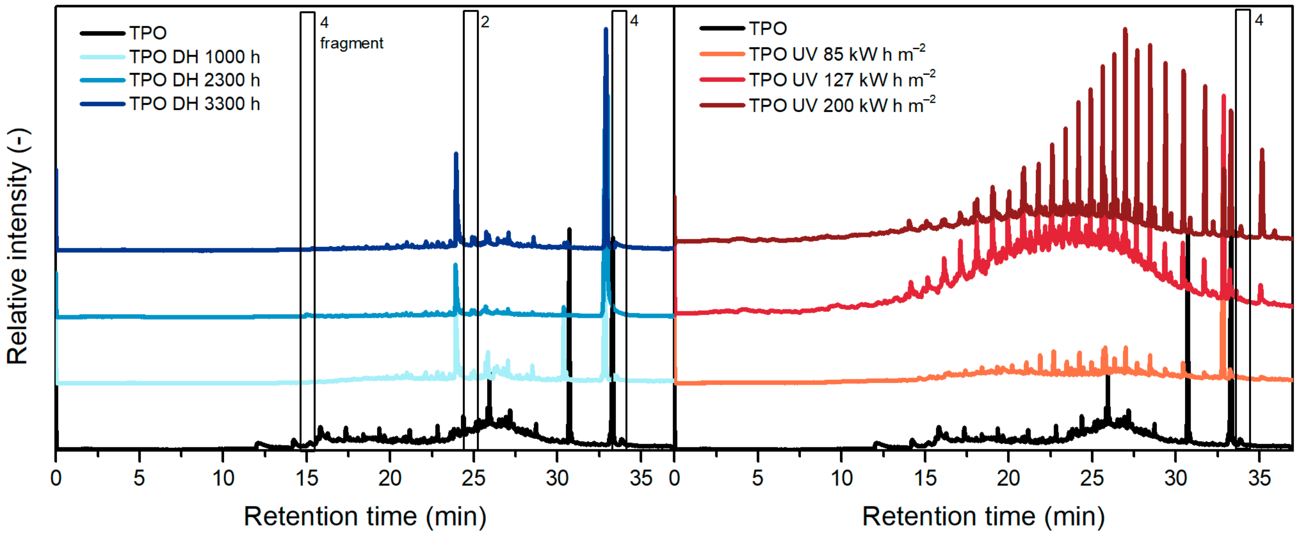

Figure S2. Chromatograms of TPO samples exposed to DH test (left) and UV test (right).

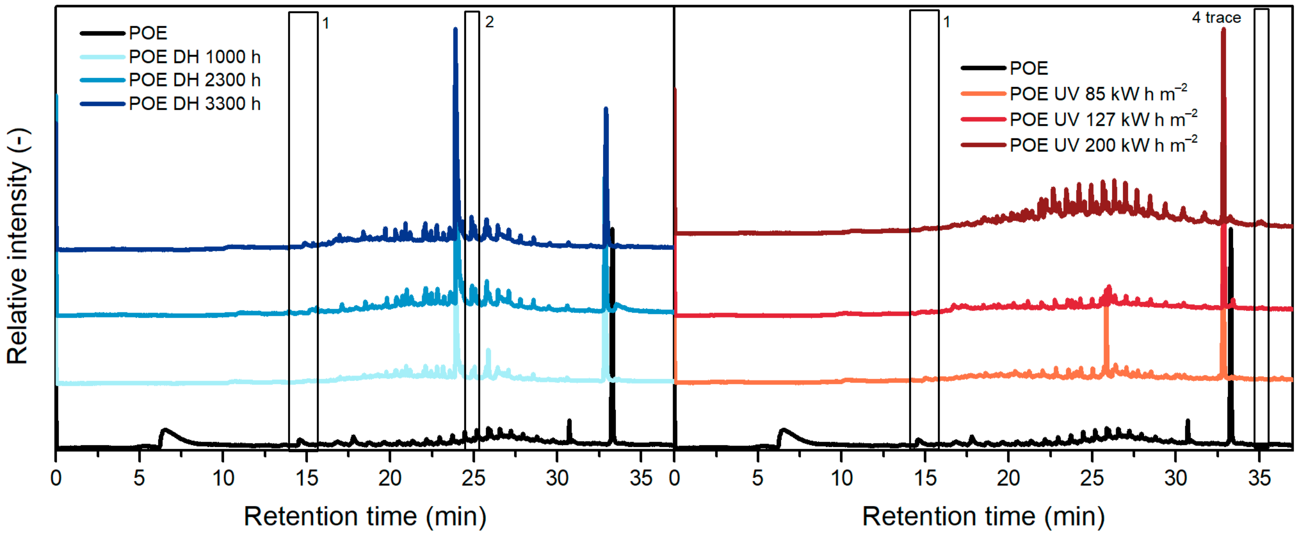

Figure S3. Chromatograms of POE samples exposed to DH test (left) and UV test (right).

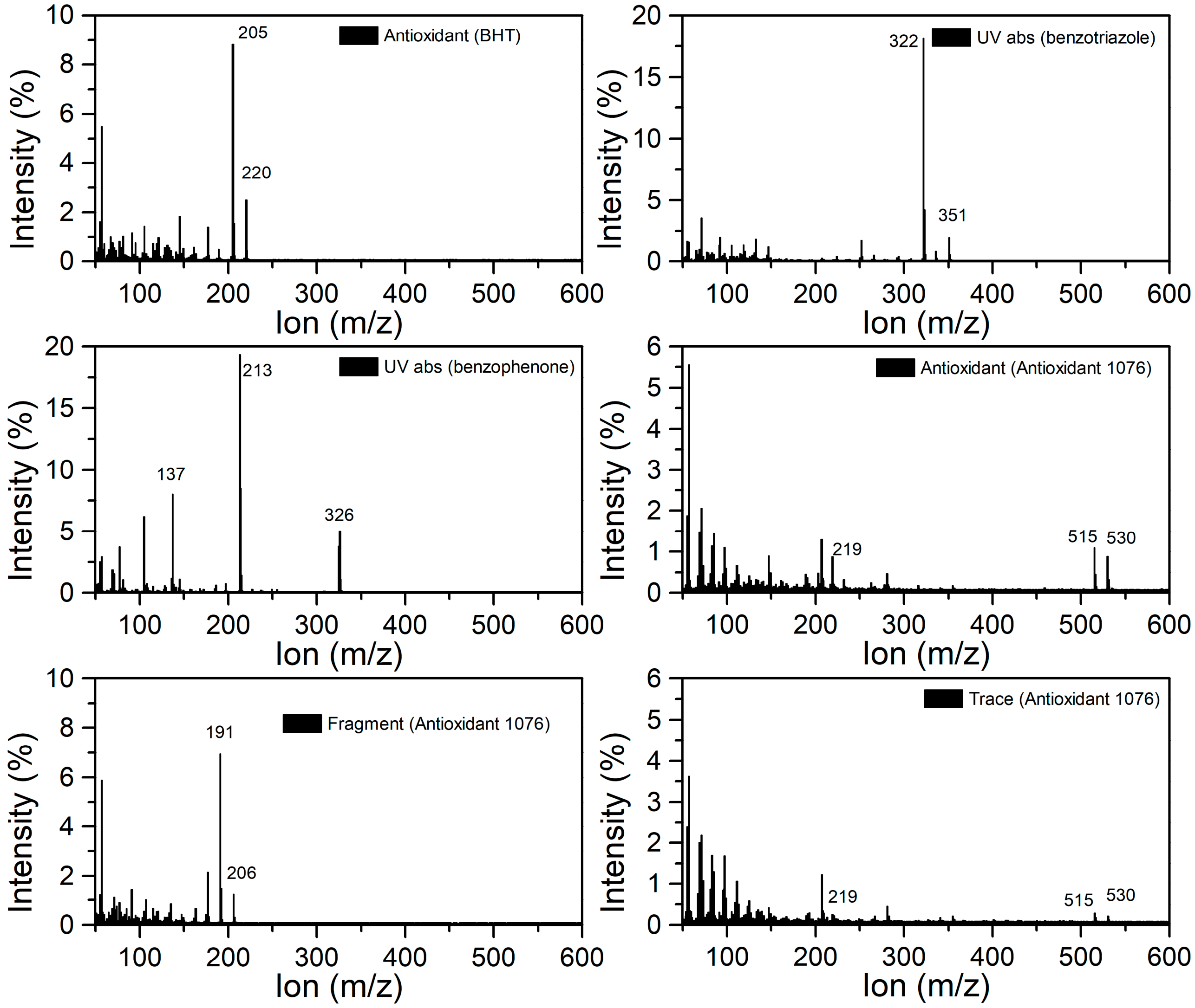

Figure S4. Mass spectra of the additives detected by means of TD-GC/MS.
